# Exosome-mediated dual drug delivery of curcumin and methylene blue for enhanced cognitive function and mechanistic elucidation in Alzheimer’s disease therapy

**DOI:** 10.3389/fcell.2025.1562565

**Published:** 2025-03-24

**Authors:** Dongxin Yang, Zhuofen Deng, Hai Zhou, Qingshang Zhang, Xibing Zhang, Jun Gong

**Affiliations:** ^1^ Central Laboratory of YunFu People’s Hospital, YunFu Key Laboratory of Brain Diseases Research, Yunfu, Guangdong, China; ^2^ Department of Pharmacy, YunFu People’s Hospital, Yunfu, Guangdong, China; ^3^ Department of Orthopedics, YunFu People’s Hospital, Yunfu, Guangdong, China

**Keywords:** Alzheimer’s disease, exosomes, curcumin, methylene blue, Tau

## Abstract

Alzheimer’s disease (AD) is one of the neurodegenerative disorders, characterized by complex pathogenic mechanisms, including the deposition of beta-amyloid protein and hyperphosphorylation of Tau protein. There is currently a lack of effective therapeutic approaches for AD treatment. The aim of this study was to design exosomes (EXO) as a specifically designed carrier able to carry Curcumin (Cur) and Methylene Blue (MB) to improve cognitive function and to elucidate its underlying mechanisms. Our study results indicated that EXO-Cur+MB inhibited Tau protein phosphorylation by activating the AKT/GSK-3β pathway, while reversing cognitive dysfunction in AD mice by reducing apoptosis induced by okadaic acid (OA). Thus, our results suggested that EXO-Cur+MB would be of potential use for the treatment of AD.

## 1 Introduction

Alzheimer’s disease (AD) is a progressive neurodegenerative disorder characterized by extracellular deposition of amyloid plaques and intracellular formation of neurofibrillary tangles. These pathological changes are closely associated with synaptic dysfunction and neurochemical deficits (Kumar et al., 2018). As AD progresses, patients experience a gradual decline in memory, cognition, and behavioral abilities, ultimately leading to a complete loss of autonomy (Atri, 2019). With the aging population, the incidence of AD is expected to rise significantly in the coming years (Brazaca et al., 2020). Existing research highlights core pathological mechanisms of AD, including hyperphosphorylation of Tau protein, accumulation of amyloid-beta plaques, neuronal apoptosis, and oxidative stress. However, the complex interactions between these factors limit the effectiveness of single-target therapies. Recent advances in neurobiology and neuropharmacology have led to the development of several promising therapeutic strategies in preclinical studies (Kumar et al., 2018). Nevertheless, current pharmacological treatments, such as acetylcholinesterase inhibitors, demonstrate limited efficacy across different stages of AD (Popp and Arlt, 2011). Therefore, there is an urgent need to identify more effective treatment options to address the cognitive and behavioral symptoms of AD patients.

Curcumin (Cur) is a natural polyphenol with notable anti-inflammatory, antioxidant, and neuroprotective properties. Cur has been shown to slow AD progression through multiple mechanisms, including the inhibition of Tau protein hyperphosphorylation, the reduction of amyloid-beta accumulation, and the modulation of cell signaling pathways (Ma et al., 2013). For example, Cur derivatives have been found to regulate the aggregation state of brain-derived Tau oligomers, consequently leading to a reduction neurotoxicity (Lo Cascio et al., 2020). Additionally, Methylene Blue (MB), a dye that has been widely used in a range of different fields, has shown significant potential in addressing AD as a neuroprotective agent. Studies have demonstrated that MB prevented Tau protein hyperphosphorylation and improved cognitive deficits and behavioral abnormalities in AD patients (Spires-Jones et al., 2014; Sun et al., 2016). In murine models, MB significantly reduces brain edema and neuronal death while improving neurological function (Zhao et al., 2016). Clinical trials have associated MB usage with improved cognitive function, suggesting its potential application in AD therapy (Zakaria et al., 2015). Despite the neuroprotective effects of Cur and MB, their therapeutic potential is limited by poor bioavailability, rapid metabolism, and difficulty in crossing the blood-brain barrier (BBB). Therefore, drug delivery methods require further optimization to improve their efficacy and bioavailability in the brain.

Exosomes (EXO), natural nanoscale extracellular vesicles ranging from 30 to 150 nm in diameter, have emerged as a promising drug delivery system due to their excellent biocompatibility, low immunogenicity, and targeting properties (Zhou et al., 2024b). They can effectively carry various bioactive molecules, including proteins, lipids, and nucleic acids, and play an essential role in intercellular communication (Kalluri and LeBleu, 2020). The unique properties of EXO make them ideal carriers for drug delivery systems (Liu et al., 2023). Compared to conventional synthetic nanoparticles, EXO demonstrate superior targeting capabilities, enhanced cellular uptake, and more controlled drug release properties (Zhou et al., 2024a; Kanchanapally et al., 2019; Batrakova and Kim, 2015). Moreover, EXO can cross the BBB via receptor-mediated endocytosis, showcasing broad therapeutic potential for neurodegenerative diseases such as AD, Parkinson’s disease, and brain cancers (Singh et al., 2024; Nouri et al., 2024). In the biosynthesis and isolation of EXO, several techniques have been developed, including ultracentrifugation, density gradient separation, and immunoaffinity-based methods, to efficiently isolate EXO from cells and other extracellular vesicles (Monguió-Tortajada et al., 2019; Salem et al., 2020; Chen et al., 2024). These advancements have significantly improved the efficiency and controllability of EXO-based drug loading and delivery.

**FIGURE 1:**
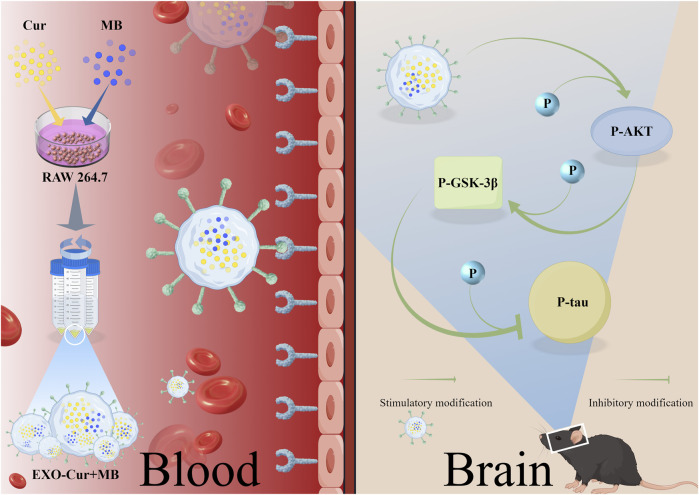
RAW264.7 cell-derived EXO loaded with Cur and MB for the treatment of AD model mice.

Given the multifactorial pathology of AD, single-agent therapies are often inadequate in addressing all aspects of the disease (Mullane and Williams, 2018). Based on the existing research into the neuroprotective properties of Cur and MB, we speculated that the combination of Cur and MB may offer a novel therapeutic strategy by enhancing neuroprotection through their potential synergistic effects. This study aims to design EXO as a specifically designed carrier able to carry Cur and MB to improve cognitive function and to elucidate its underlying mechanisms. In our study, okadaic acid (OA) was employed to induce Tau hyperphosphorylation, thereby establishing an animal model for AD (Wang et al., 2019). The interaction between the lymphocyte function-associated antigen 1 (LFA-1) and endothelial intercellular adhesion molecule 1 (ICAM-1) plays a key role in the immune response and cell adhesion process. This interaction is particularly important in the BBB, which promotes migration of immune cells to the brain. Unactivated macrophage EXO act as natural nanocarriers that can utilize this interaction to cross the BBB and deliver therapeutic drugs to the brain (Wang et al., 2019). The study aimed to assess the efficacy of EXO-Cur+MB in mitigating OA-induced learning and memory impairments and to elucidate its role in modulating AKT/GSK-3β-mediated Tau phosphorylation within the context of OA-induced Tau-phosphorylated AD-like mouse models. Additionally, we explored the potential of OA to mitigate cognitive deficits in mice through its effects on apoptosis. Our hypothesis and experimental program are shown in Figure 1.

## 2 Materials and methods

### 2.1 Cell culture and reagents

The macrophage cell line, RAW 264.7 (mouse leukemic monocyte macrophage cell line), was provided by Nanfang Hospital, Southern Medical University. The murine macrophage RAW 264.7 cells were cultured in a complete growth medium consisting of Dulbecco’s Modified Eagle’s Medium (Gibco, United States) supplemented with 10% EXO-Depleted Fetal Bovine Serum (VivaCell, CHN) and 1% penicillin-streptomycin (Gibco, United States). The cells were maintained at 37°C in a humidified atmosphere with 5% CO_2_. Sub-culturing was performed once the cell confluence reached over 80%.

### 2.2 Animals

Male C57BL/6 mice, aged 5–7 weeks, were obtained from GUANGZHOU RUIGE BIOLOGICAL TECHNOLOGY CO., LTD. and were kept in the Animal Center of Central laboratory of Yunfu People’s Hospital. The mice were maintained in groups of three per cage under standard laboratory conditions (temperature 24°C ± 1°C, humidity ranging from 60% to 70%, and a 12-h light/dark cycle) with free access to food and water. All animal procedures were conducted in strict accordance with the Animal Ethics Procedures and Guidelines of the People’s Republic of China. The study protocol was approved by the Institutional Review Board of Yunfu People’s Hospital, ensuring compliance with ethical standards to safeguard the health and welfare of the mice throughout the experimental procedures (Supplementary Data Sheet 1).

### 2.3 Collection and characterization of EXO series products

Drug-loaded EXO were prepared using co-incubation of drugs with cells and gradient centrifugation. Cur (Macklin, CHN) was dissolved in dimethyl sulfoxide (Servicebio, CHN) to prepare a 1 mg/mL stock solution and then diluted to 40.00 μg/mL with EXO-free Dulbecco’s Modified Eagle’s Medium complete medium. MB (Macklin, CHN) was dissolved in ultrapure water to prepare a 1 mg/mL stock solution and then diluted to 34.73 μg/mL with EXO-free Dulbecco’s Modified Eagle’s Medium complete medium. Macrophage RAW 264.7 cells were treated with Cur (40.00 μg/mL) and MB (34.73 μg/mL) for 24 h, after which the culture media were collected. The media were filtered through a 0.22 μm microporous membrane, and then centrifuged at 15,000 r/min for 30 min at 4°C to remove cellular debris. The supernatant was further centrifuged at 60,000 r/min for 2.5 h, and the resulting supernatant was discarded. The pellet was resuspended in phosphate-buffered saline to obtain concentrated drug-loaded EXO particles.

The drug-loaded EXO were characterized using a transmission electron microscope (TEM) (HITACHI, JPN), particle size analysis, and detection of characteristic proteins. Appropriate amounts of EXO, EXO-Cur, EXO-MB, and EXO-Cur+MB solutions were evenly dispersed and dropped onto carbon film-coated copper grids for morphological observation by TEM. The well-mixed EXO, EXO-Cur, EXO-MB, and EXO-Cur+MB solutions were placed in measurement dishes, and the particle size was measured by dynamic light scattering (ZSU3100, Malvern Panalytical Limited, UK). Western blot (WB) was used to detect the expression of CD63, Alix, Calnexin, and LFA-1.

Cur was dissolved in methanol, and MB was dissolved in ultrapure water, respectively, to prepare stock solutions of 10 μg/mL and 20 μg/mL. An aliquot of each stock solution was precisely measured and diluted with the corresponding solvent to obtain a series of concentrations: 0.25, 0.5, 1.0, 2.5, 5.0, and 10 μg/mL for Cur, and 0.5, 1.0, 2.5, 5.0, 10, and 20 μg/mL for MB. The corresponding solvents were used as blank controls. The absorbance values of Cur solution at 425 nm (D'Souza and Devarajan, 2013) and MB solution at 664 nm (Patil et al., 2023) were determined, and standard calibration curves were plotted. The absorbance of the naïve EXO was also measured and used as a reference to remove background interference. Linear regression analysis was performed using absorbance (y) versus concentration (x). Simultaneously plot the absorbance curve of phosphate-buffered saline -resuspended concentrated EXO particles, and determine the drug loading content based on the standard curves of Cur solution and MB solution.

### 2.4 Induction of *in vivo* AD model

In this study, an AD model was established using SPF-grade C57BL/6 mice. The mice were anesthetized with 2% isoflurane gas and fixed on a stereotaxic instrument for small animals (RWD69100, CHN). The hair on the head was shaved, the scalp was incised, and the area was disinfected with 75% alcohol. Based on the mouse brain stereotaxic atlas, the coordinates for the injection site in the right hippocampus were determined (bregma −2.18 mm, lateral −1.5 mm, vertical depth −1.8 mm). A hole was drilled at the determined site, taking care to avoid damage to the meninges. A microsyringe pump was used to inject 1.5 μL of 0.2 μM OA (Upstate, United States) solution at a rate of 0.3 μL/min over approximately 5 min. After the injection, the needle was left in place for 2 min to ensure drug diffusion before being slowly withdrawn. Penicillin powder was applied to the drilled hole to prevent infection, and the incision was sealed with glass ionomer cement. The mice were monitored postoperatively, with adequate food and water provided. Five days after surgery, Morris Water Maze behavioral tests were performed. The mice were randomly and equally divided into five groups (n = 3), and continuously intraperitoneally injected with different drugs for 7 days. Sham group (normal control mice) and OA (blank model control mice) group were treated with saline (150 μL per day), respectively. OA + Cur group, Cur-treated model mice, were treated with Cur solution (5 μg/mL, 150 μL per day). OA + MB group, MB-treated model mice, were treated with MB solution (10 μg/mL, 150 μL per day). OA + EXO-Cur+MB group, EXO-Cur+MB-treated model mice, were treated with EXO-Cur+MB solution (150 μL per day).

### 2.5 Morris Water Maze test

The Morris Water Maze (YUYAN YAN-MWMR, CHN) test was used to evaluate spatial learning and memory abilities in AD mice. The experimental period was set for 6 days, with the first 5 days dedicated to the place navigation test and the sixth day for the spatial probe test. The experiment was conducted in a circular pool with a diameter of 120 cm and a height of 40 cm. The water depth was maintained at 30 cm, and non-toxic titanium dioxide was added to make the water opaque white, enhancing contrast with the black C57BL/6 mice. The pool was divided into four equal quadrants, and an 8 cm × 8 cm transparent platform was placed in the center of one quadrant. The platform was submerged 1 cm below the water surface and remained in a fixed position throughout the experiment.

To reduce stress responses in the mice, the laboratory environment was maintained at 22°C–25°C. During the first 2 days of the guided learning phase, mice were allowed to swim freely in the water for up to 90 s before being promptly guided to the platform, where they stayed for 15 s while observing a yellow signboard placed opposite. The next 4 days were for the place navigation test, where once the mice found the platform, they were immediately removed from the pool, and the number of platform crossings and the percentage of time spent in the target quadrant were recorded.

On the sixth day, a spatial probe test was conducted, in which the platform was removed, and the mice were allowed to swim freely in the pool to assess their spatial memory. Metrics recorded included swimming time and path. Experimental data were automatically collected using a video tracking system.

### 2.6 Protein extraction and WB analysis in brain tissue

Brain tissue samples were minced on ice and homogenized in lysis buffer at a ratio of 150–250 μL buffer per 20 mg of tissue, containing protease and phosphatase inhibitors. The homogenate was centrifuged at 12,000 g for 15 min at 4°C, and the supernatant was collected for protein quantification using the Bicinchoninic Acid Assay. Proteins were separated by Sodium Dodecyl Sulfate-Polyacrylamide Gel Electrophoresis and subsequently transferred onto Polyvinylidene fluoride membranes using a semi-dry transfer method at 25 V for 30 min. The Polyvinylidene fluoride membranes were blocked with 5% non-fat milk at room temperature for 1 h and then incubated with primary antibodies overnight at 4°C. After washing, the membranes were incubated with Horseradish Peroxidase-conjugated secondary antibodies at 37°C for 1 h. Finally, protein bands were visualized using an enhanced chemiluminescence system, and the expression levels of the protein bands were analyzed using digital imaging tools.

### 2.7 Statistical analysis

Statistical analysis was performed with data expressed as mean ± standard deviation. Student’s t-test and one-way ANOVA were used to assess differences between groups. All analyses were conducted using SPSS and GraphPad Prism software, with statistical significance defined as *P* < 0.05.

## 3 Results

### 3.1 Characterization of EXO series products

**FIGURE 2:**
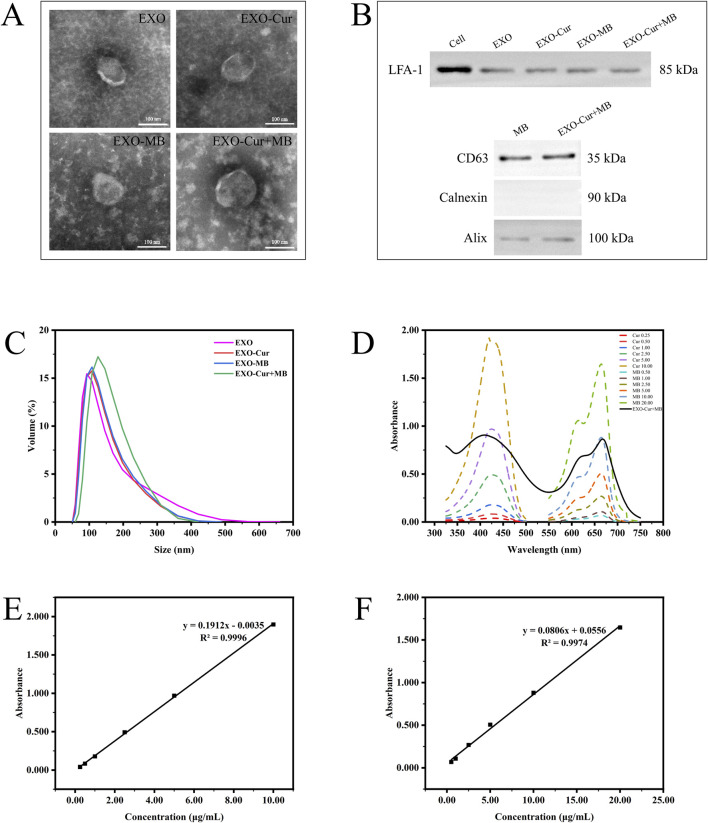
The characterization of EXO series products: **(A)** Morphology observed by TEM. **(B)** WB analysis of LFA-1, CD63, Calnexin, and Alix in RAW264.7 cells and EXO series products. **(C)** Particle size distribution measured by dynamic light scattering. **(D)** Absorbance of Cur and MB solutions at different concentrations, along with the absorbance of EXO-Cur+MB solution. **(E, F)** Standard curves of Cur and MB solutions.

As shown in Figure 2A, TEM revealed that all EXO series products displayed typical structures. The dynamic light scattering analysis indicated that the peak sizes of EXO, EXO-Cur, EXO-MB, and EXO-Cur+MB were 92.89 nm, 108 nm, 108 nm, and 125.6 nm, respectively (Figure 2C). WB analysis detected the positive markers Alix and CD63 for EXO, while the negative marker Calnexin was not detected. Additionally, the presence of LFA-1 was confirmed, suggesting that LFA-1 on the surface of EXO series products can specifically interact with ICAM-1 on brain microvascular endothelial cells of the BBB, facilitating the passage of EXO-Cur+MB through the BBB (Figure 2B) (Wang et al., 2019). In our previous study, the specific marker proteins CD63 and Alix were both expressed in EXO and EXO-Cur, while Calnexin was not detected (Dongxin et al., 2024). Standard curves of Cur and MB solutions at different concentrations were generated to determine the concentration of loaded drugs in the EXO-Cur+MB solution (Figure 2D). The linear equation for the Cur solution was y = 0.1912x - 0.0035, *R* ^2^ = 0.9996 (Figure 2E), and for the MB solution, y = 0.0806x + 0.0556, *R* ^2^ = 0.9974 (Figure 2F). In 150 μL of EXO-Cur+MB solution, 0.70 μg of Cur and 1.49 μg of MB were loaded.

### 3.2 Effects of EXO-Cur+MB on spatial learning and memory in OA-induced AD model mice

**FIGURE 3:**
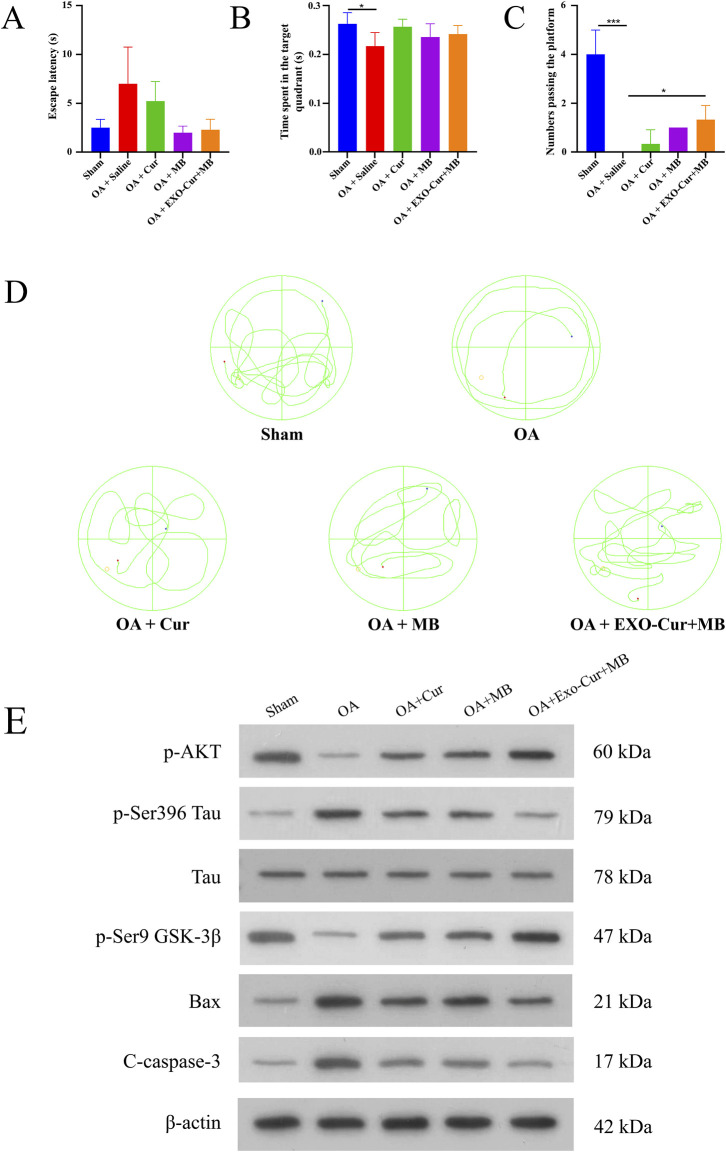
Therapeutic effects *in vivo*. **(A)** The escape latency (time used to find the platform) of mice for different times. **(B)** The time that the mice spent in the target quadrant. **(C)** The platform crossing number in the spatial probe test. **(D)** Representative swimming trajectories of mice. **(E)** WB analysis in mice hippocampus. *P < 0.05, ***P < 0.001.

The Morris Water Maze test is a widely used measure of spatial learning and memory. The results indicated that, on the fifth day, the OA group exhibited a longer escape latency than the Sham group (Figure 3A). On the sixth day, the OA group crossed the platform fewer times and spent significantly less time in the target quadrant compared to the Sham group, indicating impaired learning and memory in the OA group (Figure 3B). However, the EXO-Cur+MB group demonstrated shorter escape latencies and a higher number of platform crossings, which indicated improved spatial memory the AD model mice treated with EXO-Cur+MB (Figure 3C). These results suggested that EXO-Cur+MB enhanced learning and memory in the OA-induced AD model mice (Figure 3D).

### 3.3 Extraction of brain tissue proteins and subsequent WB analysis

To elucidate the mechanism by which EXO-Cur+MB modulates Tau protein phosphorylation, WB analysis was performed (Figure 3E). GSK-3β, a kinase implicated in Tau phosphorylation, is known to dephosphorylate at Ser9 upon activation, thereby promoting Tau phosphorylation. Our results revealed a significant decrease in GSK-3β phosphorylation at Ser9 in the OA group compared to the Sham group, suggesting OA-induced activation of GSK-3β. However, EXO-Cur+MB treatment led to a significant increase in GSK-3β phosphorylation at Ser9 relative to the OA group, indicating that EXO-Cur+MB suppressed OA-induced GSK-3β activation.

AKT, an upstream regulator of GSK-3β, suppresses GSK-3β activation by enhancing Ser9 phosphorylation. AKT phosphorylation was significantly diminished in the OA group compared to the Sham group, suggesting to OA-induced AKT inactivation. In contrast, EXO-Cur+MB treatment significantly elevated AKT phosphorylation levels compared to the OA group, suggesting that EXO-Cur+MB enhanced AKT activation. Moreover, EXO-Cur+MB treatment resulted in upregulated GSK-3β phosphorylation at Ser9 and downregulated Tau phosphorylation at Ser396 compared to both the free Cur and free MB groups, indicating that EXO-Cur+MB more effectively inhibited Tau hyperphosphorylation by suppressing the AKT/GSK-3β/Tau pathway.

Given that apoptosis in the hippocampal tissue can also lead to cognitive dysfunction in AD mice, we assessed the expression of apoptotic markers Cleaved Caspase-3 and Bax. The OA group exhibited higher levels of these markers than the Sham group, indicating increased apoptosis following OA induction. EXO-Cur+MB treatment significantly reduced the expression of Cleaved Caspase-3 and Bax compared to the OA group. Furthermore, the EXO-Cur+MB demonstrated a better anti-apoptotic effect when compared to the free Cur and free MB groups. These results suggested that EXO-Cur+MB suppressed Tau protein hyperphosphorylation by targeting the AKT/GSK-3β/Tau pathway and also inhibited apoptosis induced by OA in AD model mice.

## 4 Discussion

This study aimed to investigate the therapeutic potential of EXO-mediated co-delivery of Cur and MB in an AD mouse model. The results showed that EXO-Cur+MB significantly improved cognitive deficits and alleviated pathological features of AD, mainly by inhibiting Tau hyperphosphorylation and suppressing apoptosis in hippocampal neurons. We provide an in-depth discussion of these findings in the context of current literature, highlighting mechanistic insights, potential implications for AD treatment, and the broader scope of neurodegenerative disease therapies.

A pivotal discovery of this study is the enhanced efficacy of EXO-Cur+MB compared to Cur or MB alone, especially in targeting the AKT/GSK-3β/Tau signaling axis. GSK-3β is a well-established kinase involved in Tau hyperphosphorylation, a central event in AD pathology that promotes neurofibrillary tangle formation and neuronal dysfunction. Our results indicated that EXO-Cur+MB treatment attenuates GSK-3β activation and promotes AKT phosphorylation, which inhibits GSK-3β activity via Ser9 phosphorylation. Utilizing EXO as a co-delivery vehicle could effectively compensate for the physicochemical limitations of Cur and MB, thereby enhancing their therapeutic efficacy in AD treatment. Notably, EXO themselves may exert inherent biological functions that synergistically interact with the encapsulated agents, potentially creating a combined therapeutic effect against AD (Wang et al., 2019). These findings highlighted the therapeutic advantage of delivering drug combinations via EXO in addressing AD’s multifactorial pathology, which involves complex signaling pathway interactions.

Although their demonstrated neuroprotective properties, Cur and MB face limited bioavailability, rapid metabolism, and difficulties in crossing the BBB (Mottola et al., 2021). EXO, as naturally occurring extracellular vesicles, efficiently cross the BBB and deliver therapeutic agents directly to target cells with high specificity. Moreover, EXO inherently protect their cargo from enzymatic degradation and extend systemic circulation time, further contributing to the observed therapeutic benefits (Lai et al., 2013). The therapeutic efficacy of the drug-loaded EXO may be attributable to this phenomenon. These properties make EXO a versatile and promising platform for delivering combination therapies in neurodegenerative diseases, where targeting multiple pathological processes is often necessary for effective intervention.

Another critical aspect of our study is the demonstrated anti-apoptotic effect of EXO-Cur+MB in the hippocampal region of AD mice. Apoptosis, driven by oxidative stress and mitochondrial dysfunction, is a major contributor to neuronal loss in AD. Inhibiting apoptosis is crucial for preserving cognitive function. Our findings showed that EXO-Cur+MB significantly reduced the expression of apoptotic markers, including cleaved caspase-3 and Bax, compared to the OA group. This anti-apoptotic effect may be due to the combined antioxidative properties of Cur and MB, which effectively mitigated oxidative damage, a key driver of apoptosis in AD. Cur has been reported to exert potent antioxidative effects by scavenging reactive oxygen species and modulating antioxidant enzyme activity (Mazzanti and Di Giacomo, 2016; Begum et al., 2008), while MB improved mitochondrial function and reduced oxidative stress (Wen et al., 2011; Atamna et al., 2007). Interestingly, the ability of EXO-Cur+MB to reduce apoptosis was more pronounced than that of either agent alone, highlighting the potential of combinatorial delivery to exert additive or synergistic effects on neuronal survival. These results provide mechanistic insights into how EXO-Cur+MB addresses Tau pathology and protects against neuronal cell death, which is essential for maintaining cognitive resilience in AD. Despite these promising outcomes, this study has limitations. The OA-induced AD model does not fully replicate human AD. Future studies should use transgenic models for better evaluation (Ashe and Zahs, 2010). Additionally, scaling up the production of EXO presents significant challenges, which must be addressed before clinical translation (Lötvall et al., 2014). Advances in EXO isolation and quality control will be crucial for ensuring the reliability and safety of these therapeutic agents.

## 5 Conclusion

In conclusion, this study demonstrated that EXO-mediated co-delivery of Cur and MB represented a promising multifaceted therapeutic strategy for AD. The co-delivery system, EXO-Cur+MB, effectively targets multiple pathological aspects of AD, including Tau hyperphosphorylation, and neuronal apoptosis, ultimately improving cognitive function in an AD mouse model. EXO-Cur+MB leverages the natural ability of EXO to cross the BBB and target specific cells, thereby improving therapeutic efficacy. These findings suggest that EXO-based combination therapies may provide a comprehensive approach to the multifactorial pathology of AD and could potentially be extended to other neurodegenerative diseases. Future research is warranted to validate these results in more sophisticated animal models and clinical trials, ultimately laying a solid foundation for innovative treatments for AD and related disorders.

## Data Availability

The datasets presented in this study can be found in online repositories. The names of the repository/repositories and accession number(s) can be found in the article/Supplementary Material.
