# Supplementary material for: Exosome-mediated dual drug delivery of curcumin and methylene blue for enhanced cognitive function and mechanistic elucidation in Alzheimer’s disease therapy
Source: Front Cell Dev Biol. 2025 Mar 24;13:1562565. doi: 10.3389/fcell.2025.1562565 (PMC11973305; doi:10.3389/fcell.2025.1562565)
Supplement: Supplementary file 1 [file DataSheet1.PDF]

云浮市人民医院医学伦理委员会
伦理审查批件

批件号：2023A065　　　　　　　　　审查日期：2023 年 6 月 20 日

地址：广东省云浮市环市东路 120 号　邮编：527300　　电话：0766-8810810

<table>
<tr><td>项目名称</td><td colspan="3">负载圆偏振荧光探针和亚甲基蓝的外泌体对阿尔茨海默病的早期诊断及治疗作用研究</td></tr>
<tr><td>申办单位</td><td colspan="3">云浮市人民医院</td></tr>
<tr><td>本院承担科室</td><td>医务科</td><td>主要研究者</td><td>宫俊</td></tr>
<tr><td>审查类别</td><td colspan="3">☑初始审查
☐复审(☐再审 ☐修正案审查 ☐跟踪审查 ☐严重不良事件审查 ☐违背方案审查 ☐暂停或终止研究审查 ☐结题审查)</td></tr>
<tr><td>审查方式</td><td colspan="3">☐会议审查　☑简易程序审查　☐紧急审查</td></tr>
<tr><td>审查文件清单</td><td colspan="3">见附件 1</td></tr>
<tr><td>审查委员名单</td><td colspan="3">[illegible]　[illegible]</td></tr>
<tr><td rowspan="3">投票结果</td><td colspan="3">伦理委员共 17 名，本次参加会议委员 2 名</td></tr>
<tr><td>同意 2 票</td><td>修改后同意 0 票</td><td>修改后再审 0 票</td></tr>
<tr><td>不同意 0 票</td><td>暂停或终止研究 0 票</td><td></td></tr>
</table>

审查意见：

该项目涉及使用实验动物，根据《实验动物管理条例》(1988 年国家科委 2 号令)、《广东省实验动物管理条例》（2010 年）、《药物临床试验质量管理规范》（2020 年）、《药物临床试验伦理审查工作指导原则》（2019 年）、《医疗器械临床试验质量管理规范》（2022 年）、《中医药临床研究伦理审查管理规范》（2010 年），世界医学会《赫尔辛基宣言》等国际国内通用伦理原则，经本伦理委员会审查，同意按所批准的临床研究（试验）方案等材料开展本项研究，并严格按照注意事项执行。

注意事项：

1. 请遵循伦理委员会批准的方案开展临床研究，严格选用符合要求的合格动物进行实验，科学合理使用、保护和善待动物。
2. 请遵守《药物临床试验质量管理规范》《医疗器械临床试验质量管理规范》《医疗技术临床应用管理办法》《干细胞临床研究管理办法(试行）》等相关规定。
3. 涉及人类遗传资源出口或者按照国家规定必须经有关部门专项审批的内容，均需在项目执行前向有关部门申报并获得批准。
4. 研究开始前，请申请人完成临床试验注册及备案。

<table>
<tr><td colspan="2">申请人提交修正案审查申请，经伦理委员会重新审查，获得批准后执行。
6. 发生严重不良事件，请申请人于发现 24 小时内提交严重不良事件报告。
7. 出现任何可能显著影响试验进行或影响受试者风险的情况及新信息时，须及时报告伦理委员会。
8. 定期/年度跟踪审查项目，无论研究开始与否，请年度/定期跟踪审查截止日期前 1 个月提交研究进展报告；申办者应当向组长单位伦理委员会提交各中心研究进展的汇报总结；如伦理审查批件有效期到期，需要申请延长批件的有效期，应通过研究进展报告申请。
9. 试验过程中，如有不依从/违背方案的情况，请及时提交不依从/违背方案报告。
10. 暂停或提前终止临床研究，请及时提交暂停/终止研究报告。
11. 完成临床研究，须及时向伦理委员会提交结题报告。
12. 不同意或终止或暂停项目，批件发出后 2 周内可向伦理委员会就有关事项做出解释或提出申诉。</td></tr>
<tr><td>年度/定期跟踪审查频率：</td><td>半年。伦理委员会有权根据研究实际进展情况改变跟踪审查频率。</td></tr>
<tr><td>伦理审查批件有效期/截止日期：</td><td>12 个月/ 2024 年 6 月 20 日。
（逾期未实施，伦理审查批件自行废止）</td></tr>
<tr><td>主任委员/被授权的副主任委员签名</td><td>[illegible]</td></tr>
<tr><td>日期</td><td>2023年6月21日</td></tr>
<tr><td>伦理委员会</td><td>云浮市人民医院伦理委员会（盖章）</td></tr>
</table>
